# LESSONS LEARNED AND NEXT STEPS FOR USER-CENTERED DESIGN WITH OLDER ADULTS AND CAREGIVERS

**DOI:** 10.1093/geroni/igae098.2067

**Published:** 2024-12-31

**Authors:** Jennifer Portz, Sara Czaja

**Affiliations:** Chair; Discussant

## Abstract

Digital health can uniquely address various healthcare challenges, yet its’ effectiveness relies heavily on factors such as usability, acceptability, and inclusivity. In this symposium, we present findings from three pioneering projects aimed at developing and refining digital health interventions for older adults and their care partners, tailored to specific populations and healthcare needs. Each project employs a user-centered design (UCD) approach to address unique challenges and opportunities in digital health intervention development. From designing dyadic interventions for advanced dementia to improving care coordination for rural older adults and integrating social support in palliative care, these projects offer valuable insights into developing meaningful, accessible, and person-centered digital health solutions across diverse healthcare landscapes. The final presentation outlines lessons learned from four remote co-design processes for dementia care partner interventions. Co-design, involving an active contribution from older adults and partners, fosters usable, relevant solutions, yet challenges in implementing co-design warrant guidance. Lastly, our Discussant, Dr. Sara Czaja, a world-renown expert in aging-related technology, will lead a conversation highlighting the benefits of UCD, the challenges and limitations of the approach, and innovative solutions for fostering the engagement of older adults and their care partners in the digital health design process.

